# Enhanced recellularization by using albumin coating with roller bottle cell culture

**DOI:** 10.1016/j.reth.2023.10.002

**Published:** 2023-10-31

**Authors:** Boyun Kim, Hyunwoo Jo, Bo Young Choi, Jina Ryu

**Affiliations:** a R&D Center, ROKIT Healthcare, Inc., Seoul, Republic of Korea; b Department of Biomicrosystem Technology, Korea University, Seoul, Republic of Korea

**Keywords:** Decellularization, Extracellular matrix, Albumin, Rotating cell culture, Recellularization

## Abstract

**Introduction:**

The decellularization and recellularization is a promising approach for tissue engineering and regenerative medicine. However, the decellularization process depletes important components like glycosaminoglycans (GAGs), affecting cell attachment and causing immunogenicity. Studies have explored various surface modification strategies to enhance recellularization.

**Methods:**

To optimize the decellularization method, we employed whole kidney perfusion and slice kidney immersion/agitation techniques. The decellularized extracellular matrix (dECM) was then analyzed using hematoxylin and eosin (H&E) staining, scanning electron microscope (SEM), and DNA quantification. To enhance cell proliferation efficiency, albumin coating and rotating culture were applied. Also, we evaluated *in vitro* blood clot formation on the albumin-coated dECM by immersing it in blood.

**Results:**

After decellularization, the unique structures of the kidney were preserved whether cellular components were removed. Subsequently, we utilized albumin coating and rotating culture for recellularization, and observed that albumin-coated dECM not only promoted high cell proliferation rates but also prevented blood clot formation.

**Conclusion:**

The albumin-coated dECM promoted cell proliferation and reduced blood clot formation *in vitro*. Also, dynamic culture condition using rotating culture allowed for improved cellular penetration into the dECM, leading to a conductive environment for enhanced tissue infiltration. This new approach suggests that the combined utilization of albumin coating and rotating culture conditions can improve the efficiency of recellularization.

## Graphical abstract

The porcine kidneys were sliced and decellularized. After decellularization, the dECM was coated with albumin to enhance cell adhesion and promote cell proliferation. Additionally, the use of rotating culture increased tissue penetration of cells and improved the efficiency of recellularization.

**Figure:**
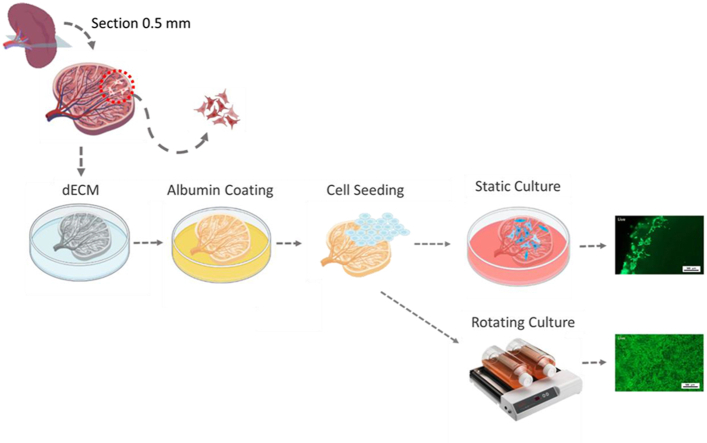

## 1 Introduction

The global demand for organ transplants has increased significantly over the past decade. However, this surge has been met with a crisis due to a shortage of organs required for successful transplants. Consequently, the number of patients on the waiting list for transplantation has significantly risen, leading to an increase in mortality rates while awaiting transplantation [1,2]. To solve these challenges, functional organs made by tissue engineering have gained attention in the field of regenerative medicine [3]. The decellularization and recellularization technologies can create functional organs that can be used for transplantation, disease modeling, drug screening, and other applications [4,5].

Since the introduction of the concept of decellularization, researchers have made attempts to remove cells from various organs and tissues using decellularization methods. Although there is no standard protocol for decellularization, it is commonly agreed upon that the decellularized extracellular matrix (ECM) should contain less than 50 ng of DNA per milligram of dried weight, with DNA fragments limited to 200 base pairs or less [[4], [5], [6]]. Furthermore, it is important to ensure the absence of cellular nuclear components within the ECM. As a result of successful decellularization, proteins such as collagen, laminin, and fibronectin are retained within the decellularized ECM [7]. However, glycosaminoglycans (GAGs) are known to be significantly depleted during decellularization process [8,9]. The depletion of adhesion molecules such as GAGs results in weak binding of repopulated cells. Uhl et al. reported that decellularized lungs led to the depletion or impairment of GAGs or side chains, which had a notable impact on the binding of growth factors to the matrix and the metabolism of lung cells. Also, the exposed vascular ECM due to cell loss induces immunogenicity and thrombogenicity after implantation [6,10]. To enhance cell attachment to dECM, there are several research on dECM surface modification strategies to enhance recellularization efficiency. Notably, Ko et al. reported the bioengineered of intact porcine livers [11]. They coated the decellularized liver scaffold with anti-endothelial cell antibody, enabling the re-establishment of vascular networks by maximizing endothelial cell coverage of vascular walls. This approach demonstrated promising results in promoting vascularization within the dECM. Another study suggested fibronectin coating on surface of decellularized aortic conduits [12]. The bio-functional protein coating increased recellularization, accelerating the *in vivo* re-endothelialization. Recently, the use of nano-graphene oxide coating was reported to improve recellularization [13]. The application of nano-graphene oxide coating enhanced the biophysical properties of decellularized liver scaffolds and reduced dECM decomposition, which is known to induce inflammation.

In this study, we observed the albumin coating not only enhanced the cell attachment, but also prevented blood clots. In addition, a rotating culture system using a roller bottle was able to improve cell viability by helping thick ECM penetration. This new approach suggests the potential use of albumin coating to improve the efficiency of recellularization.

## 2 Materials and methods

### 2.1 Optimization of decellularization process

The porcine kidney was decellularized to produce decellularized scaffolds. For whole porcine kidney decellularization, porcine kidney was perfused with Heparin containing PBS for 3 h (10 ml/min). After perfusion with heparin, Detergent 1 solution (R-003, ROKIT Healthcare, Inc., Seoul, Korea), which is based on Triton X-100, was used for decellularization at a rate of 10 ml/min for 12 h. Then, Detergent 2 solution (R-004, ROKIT Healthcare, Inc.), which is based on Sodium dodecyl sulfate (SDS), was perfused at a rate of 5 ml/min for 24 h. In the case of tissue slices, heparinized kidneys are stored at −20 °C and then prepared using a sectioning machine to have thicknesses of 0.5, 2 and 10 mm. The prepared samples were placed in a shaker and decellularized using Detergent 1 for 2 h. Afterwards, decellularization was performed for 1 day by replacing the solution with Detergent 2 every 12 h.

### 2.2 Recellularization in a roller bottle

The decellularized kidney slices were treated with 25 % albumin (A9418, Sigma-Aldrich, Saint Louis, MO, USA) in PBS overnight at 4 °C. After coating, the tissue was washed three times with PBS, then 2 × 10^4^ cells were seeded onto it. To help the cells attachment, the tissue, after cell seeding, was placed in the incubator in a static state for 30 min. Subsequently, the tissue was transferred to a roller bottle (JETBIOFIL Roller Bottles, PS TCB012001), and the cells were cultured for 3 days at a speed of 17 rpm through the roller control (NEST Standard Mini Roller, #105006, Wuxi NEST Biotechnology, Jiangsu, China).

### 2.3 Morphology observation and staining with hematoxylin and eosin (H&E)

dECM was fixed with 4 wt/vol buffered parformaldehyde, and then paraffin was prepared to be fragmented (thickness of 3 μm) using microtom (RM2135, Leica, Wetzlar, Germany). After decellularization, samples were stained with H&E (Sigma-Aldrich) according to the manufacturer's instructions. For histological images, the CaseViewer™ program was used.

#### 2.3.1 Field-emission scanning electron microscope (FE-SEM)

After conducting primary fixation using Karnovsky's fixative (2 % paraformaldehyde, 2.5 % glutaraldehyde in 0.1 Molar sodium phosphate buffer), the samples were washed three times with 0.05 M sodium cacodylate buffer at room temperature. For post-fixation, 1 % osmium tetroxide was diluted in 0.1 M sodium cacodylate buffer and the process was carried out for 1 h at 4 °C. Following three washes with distilled water, dehydration was performed at room temperature by gradually changing the ethanol (30–100 %). Subsequently, the specimen was dried and imaged using FE-SEM, SUPRA (Carl Zeiss, Oberkochen, Baden-Württemberg, Germany).

### 2.4 DNA quantification

The DNA content was measured using AccuPrep® Genomic DNA Extraction Kit (Bioneer, Daejeon, Korea) according to the manufacturer's instructions. Briefly, homogenization of tissue with mortar, then Protease and RNase were added and mixed. After complete lysing of tissue, the samples were analyzed by microplate spectrophotometer (Epoch 2, Bio Tek, Winooski, Vermont, USA).

### 2.5 Albumin coating

The albumin solution was prepared by diluting bovine serum albumin (#BASS0.1, Bovogen Biologicals, East Keilor, Victoria, Australia) in PBS. Then, dECM was immersed in the albumin solution for 6 h at room temperature. After albumin coating, the dECM was washed twice with PBS. Kidney primary cells were seeded at the density of 20,000 cells/cm^2^ and cultured on dECM in DMEM (Dulbecco's Modified Eagle Medium, #11960044, Gibco-Invitrogen, Carlsbad, CA, USA) with 10 % FBS (#16000044, Gibco-Invitrogen) and 1 % PS (#10378016, Gibco-Invitrogen) media.

### 2.6 Cell viability assay and live cell staining

Cell viability after recellularization was measured by EZ-cytotox kit (#EZ-500, Dogenbio, Seoul, Korea) according to the manufacturer's guide. In briefly, dECMs were coated with albumin then 2 × 10^4^ cells were seeded on that. For static culture, dECM were incubated in well-plate, and for rotating culture dECM was incubated in roller bottle. After 5 days incubation, cell viability was measured by ELISA. Absorbance was measured at 450 nm using an ELISA reader (#15140148, Gibco-Invitrogen). For staining, LIVE/DEAD™ Viability/Cytotoxicity Kit (#L3224, Thermo Fisher Scientific, Waltham, MA, USA) was performed, and observed by fluorescence microscope (ECLIPSE Ts2, Nikon, Tokyo, Japan).

### 2.7 Statistical analysis

All values are presented as the mean ± S.D. All experiments were performed in triplicate and compared with the control, using the *t*-test. *∗p* < 0.05, *∗∗p* < 0.01, and *∗∗∗p* < 0.001 were considered statistically significant values. All statistical analyses were performed by SigmaPlot (Systat Software, San Jose, CA, USA).

## 3 Results

### 3.1 Optimization of decellularization for whole or slice kidney

**Fig. 1:**
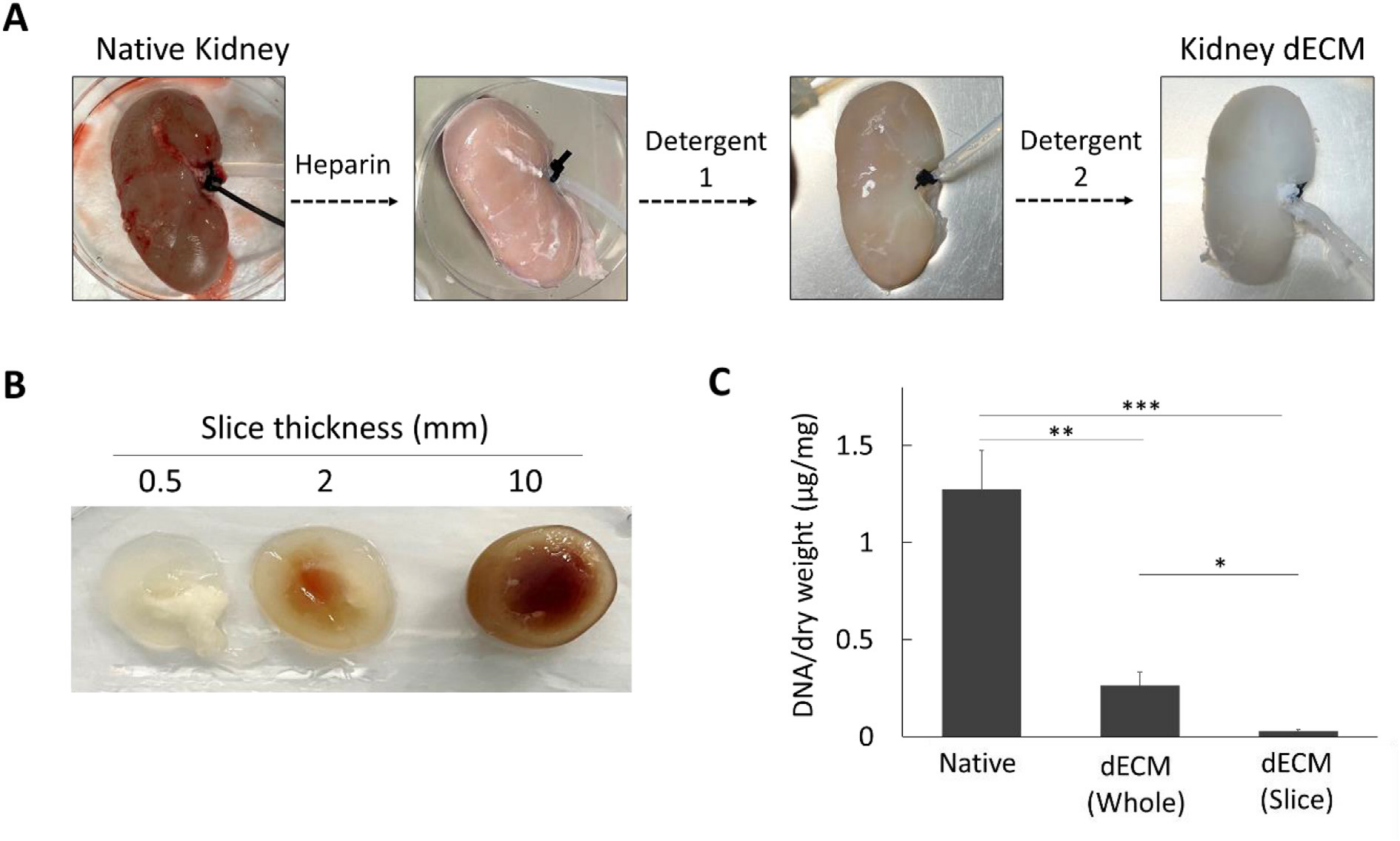
Decellularization of porcine whole or slice kidney. The porcine kidneys were decellularized using perfusion for whole kidneys and immersion/agitation for slice kidneys. (A) For the decellularization of the whole kidney, heparin and detergents were sequentially perfused following the native kidneys. (B) Slices having thicknesses of 0.5, 2, and 10 mm were immersed in detergents and agitated for decellularization. (C) The DNA contents of dECM in whole or slices kidneys were analyzed in comparison to the native kidney. ∗*p* < 0.05, ∗∗*p* < 0.01, and ∗∗∗*p* < 0.001.

To generate kidney dECM, we utilized two distinct approaches. In the first approach, we perfused the whole kidney through the arteries with detergents following administration of heparin to prevent blood clotting. The decellularization process was completed by perfusing with detergents (Fig. 1A). In the second approach, we prepared slice kidney of 0.5-, 2-, and 10-mm thickness and optimized the decellularization conditions (Fig. 1B). The decellularization process was found to be most rapid and effective when the thickness of the kidney slice was 0.5 mm. However, when the slice thickness exceeded 2 mm, the decellularization time extended to over a day. After decellularizing a slice (0.5 mm) or the whole kidney, the DNA contents were analyzed (Fig. 1C). The remaining DNA in the slice dECM was 29 ng of DNA per mg dECM, while the whole dECM contained 263 ng of DNA per mg dECM. These amounts represented a 97.7 % and 79.4 % decrease in DNA contents, respectively, compared to native kidneys (1275 ng of DNA per mg dECM). Therefore, for further study, the kidney tissue was sliced to a uniform thickness of 0.5 mm to ensure consistent and efficient decellularization.

### 3.2 Preservation of kidney structure after decellularization

**Fig. 2:**
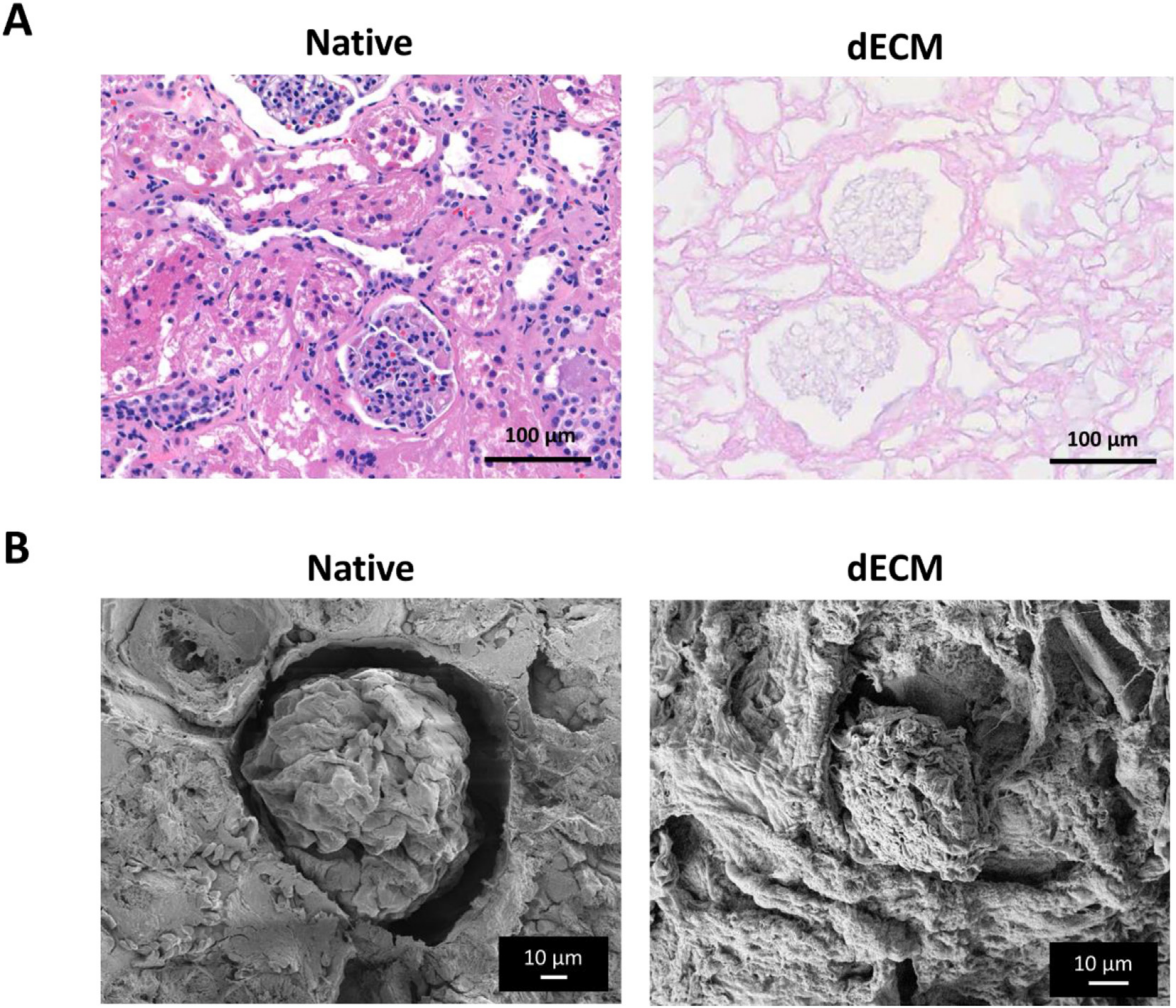
Analysis of native and dECM kidneys after decellularization. After decellularization of kidneys, the decellularized extracellular matrix (dECM) was analyzed. (A) Hematoxylin & eosin (H&E) staining showed the removal of cellular components within the dECM (A). The scale bar represents 100 μm. (B) The preservation of kidney's microarchitecture was observed by scanning electron microscope (SEM). The scale bar represents 10 μm.

After decellularizing the kidney slices, H&E staining was performed to verify the successful removal of cellular components from the dECM (Fig. 2A). The images confirmed the preservation of the glomerular structure, while revealing the removal of cells or nuclei. Additionally, scanning electron microscope (SEM) was used to further validate the preservation of the kidney's unique glomerular structure following decellularization. In Fig. 2B, the extracellular matrix (ECM) scaffold exhibited a kidney structure reminiscent of glomeruli, but no cells were observed. Therefore, these findings indicate that the decellularized kidney dECM effectively eliminated all cellular components while maintaining its original structure.

### 3.3 Albumin-coating can prevent blood clot formation on dECM

**Fig. 3:**
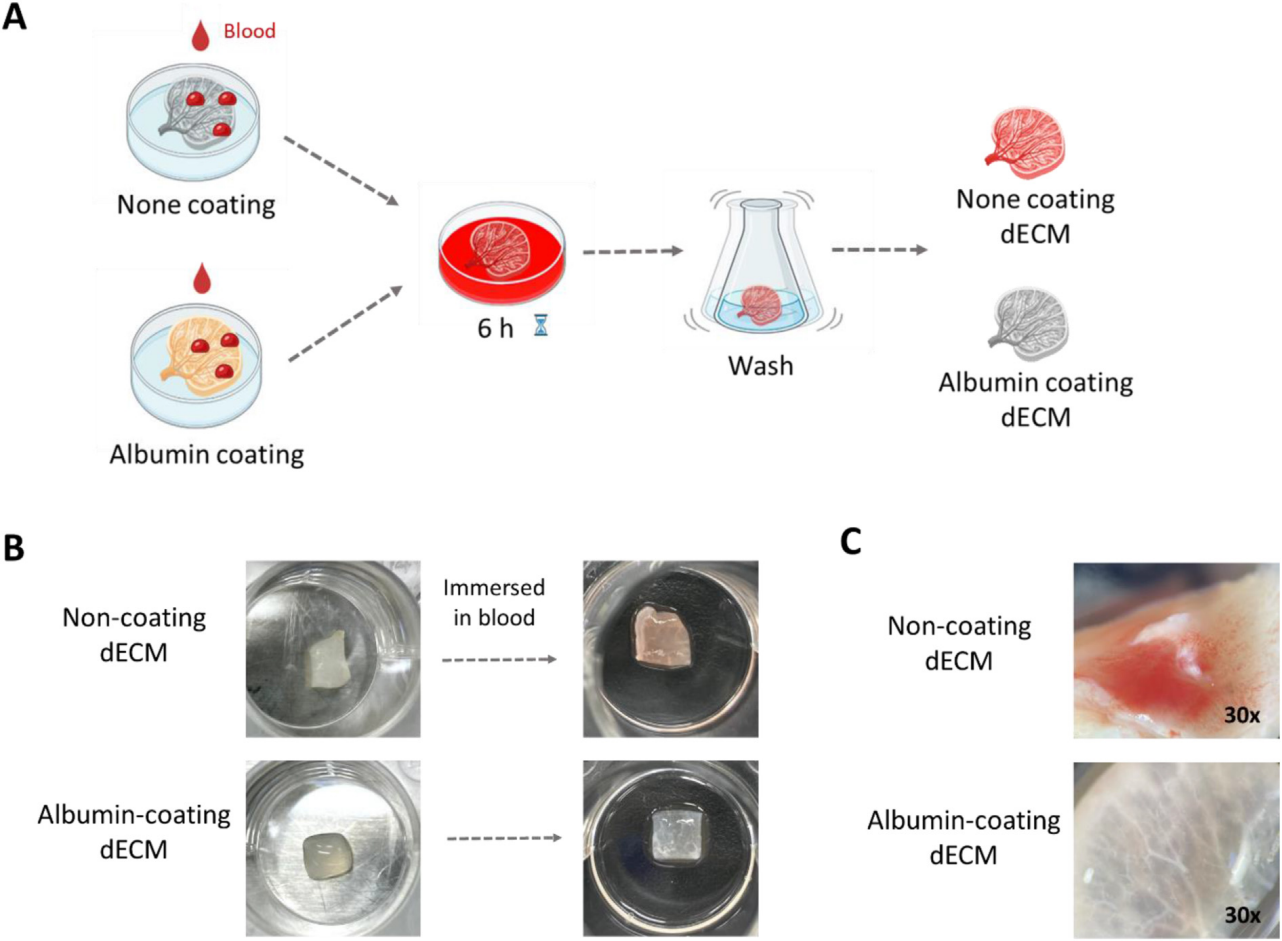
*In vitro* blood clot formation test. (A) A schematic illustration of the process. After preparing the albumin coated dECM and non-coated dECM, it was immersed in blood for 6 h and then washed. (B) The photographs of blood clotting on the surface of the dECM. (C) The dECM was observed under a microscope. 30× magnification.

We investigated the effectiveness of albumin coating in preventing blood clotting on dECM. To assess the albumin coating, dECM tissue was coated with a 25 % albumin solution diluted in PBS and then immersed in whole blood for 6 h (Fig. 3A). Non-coated dECM remained visibly red even after washing, while albumin-coated dECM showed no red coloration after washing (Fig. 3B). In addition, microscopic observations confirmed minimal thrombus formation on albumin-coated dECM (Fig. 3C). Therefore, these findings suggest that blood clots can be prevented by coating dECM with albumin.

### 3.4 Albumin coating and rotating culture enhanced recellularization

**Fig. 4:**
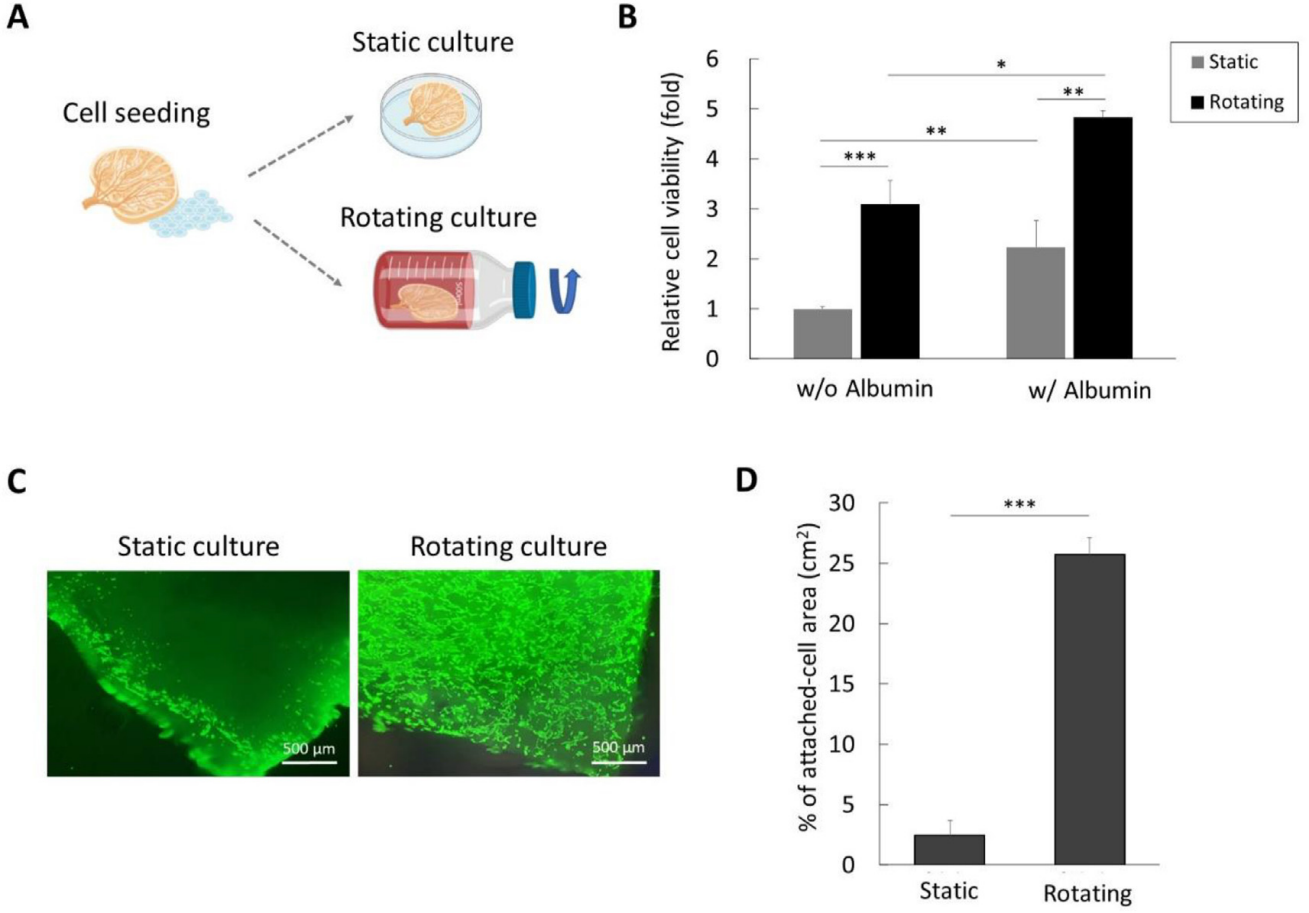
Albumin coating and rotating culture for recellularization. (A) Cells were seeded on dECM and cultured in static or rotating conditions. (B) The relative cell viability was evaluated in static or rotating cultures, without or with albumin. (C) Live cell staining was performed after recellularization with albumin, comparing static and rotating cultures. The scale bar represents 500 μm. (D) The attached-cell area was analyzed in static or rotating cultures using image J software, with calculations performed in four randomly selected areas. ∗*p* < 0.05, ∗∗*p* < 0.01, and ∗∗∗*p* < 0.001.

After decellularization, renal epithelial cells were seeded on dECM, and incubated using static or rotating culture conditions (Fig. 4A). To promote cell attachment, the tissue with cells was held stationary in the cell incubator for 30 min before initiating rotating culture. Also, albumin was used to coating materials for dECM. After 5 days of cell seeding, we examined the cell viability on dECM. In static culture, the presence of albumin coating resulted in a 2.2-fold increase in cell viability compared to the condition without albumin coating. Similarly, in rotating culture, there was 1.6-fold increase in cell viability with albumin coating compared to without albumin coating. Notably, rotating culture consistently showed higher cell viability rates regardless of the presence of albumin coating. Next, live cells were stained to confirm the distribution of cells in albumin-coated dECM (Fig. 4C). From the images, we observed that where cells only attached to the surface in static culture, rotating culture showed cell infiltration into the dECM. Fluorescence intensity of 4 random areas was calculated using image J software (Fig. 4D). The average fluorescence in the rotating culture showed approximately 10.5-fold more than those in the static culture. Therefore, it was concluded that albumin coating improved cell viability in dECM, especially when combined with rotating culture, it can increase cell infiltration into tissues and maximize cellular proliferation efficiency.

## 4 Discussion

In this study, we revealed that albumin coating and rotating cell culture enhanced recellularization by increasing cell attachment on dECM and cell viability. To enhance recellularization, the study focused on investigating the interaction with cell and ECM surface. Zelzer et al. reported that treating a hydrophilic plasma polymer with a mixture of fibronectin and albumin resulted in the highest cell adhesion on the plate surface [14]. Although albumin is known as a protein which hinders cell attachment, its application on implantable surfaces had contrary effects by promoting the adhesion and proliferation of cells [15]. Albumin, as a biocompatible and non-toxic protein, has been already used in clinical applications [[16], [17], [18]]. Using albumin as a coating material for cell attachment, the Koblinski et al. reported that the presence of albumin plays a triggering role and induces cell attachment [19]. Also, they revealed that albumin enhanced cell attachment by exposing the integrin-binding site of fibronectin. Moreover, the ECM is consisted of numerous proteins, including cell attachment protein such as fibronectin, which allows the ECM to have hydrophilic properties [20]. The cell-derived ECM exhibited hydrophilic properties with 66.8° water contact angle, creating an environment conductive to the adhesion and differentiation of cells [21]. In our study, we observed that coating albumin on ECM increased cell adhesion and viability compared to the non-coated group. It is considered that albumin assisted cells in effectively attaching to adhesive proteins present in ECM.

After transplantation of recellularized organs, the loss and removal of cells cause thrombogenesis [22]. To make transplantable organs, surface coating techniques for resolving hemocompatibility issues are necessary. Coronel-Meneses et al. reported cases where the surface coating of cardiac medical device with chemical compounds addressed problems such as platelet aggregation, thrombus formation, and bacterial infections [23]. Various biomaterials are being used for *anti*-thrombogenic surface coating. Albumin as a bio-passive coating material showed *anti*-thrombogenic effect [24]. By binding to fibrinogen/fibrin, albumin can act as an anticoagulant [25]. In fact, the administration of albumin supplementation to COVID-19 patients allowed for anticoagulant therapy [26]. The administration of albumin intravenously to COVID-19 patients enhanced hemodynamics and resulted in a reduction in the plasma concentration of D-dimer, a primary indicator of thromboembolism [27]. Our findings align with previous research, indicating that albumin coatings applied to dECM effectively mitigate thrombus formation.

These results demonstrate the potential of albumin for cell viability and thrombus prevention. However, to utilize albumin in various types of tissue scaffolds, it is crucial to optimize conditions and evaluate the impact of albumin coating on the differentiation and functionality of administered cells. Additionally, assessing the long-term effects and implantability of albumin coatings in tissue engineering and regenerative medicine necessitates further research on stability and efficacy evaluation. Nevertheless, the use of albumin as a natural and biocompatible molecule to promote cell growth and differentiation holds promising prospects in the field of regenerative medicine applications.

## Declaration of competing interest

The authors declare the following financial interests/personal relationships which may be considered as potential competing interests:

Jina Ryu reports financial support was provided by ROKIT HEALTHCARE Inc. Jina Ryu reports financial support was provided by Korean Fund for Regenerative Medicine (KFRM).
